# *GABRB2* Haplotype Association with Heroin Dependence in Chinese Population

**DOI:** 10.1371/journal.pone.0142049

**Published:** 2015-11-12

**Authors:** Yung Su Kim, Mei Yang, Wai-Kin Mat, Shui-Ying Tsang, Zhonghua Su, Xianfei Jiang, Siu-Kin Ng, Siyu Liu, Taobo Hu, Frank Pun, Yanhui Liao, Jinsong Tang, Xiaogang Chen, Wei Hao, Hong Xue

**Affiliations:** 1 Division of Life Science and Applied Genomics Center, Hong Kong University of Science & Technology, Clear Water Bay, Hong Kong, China; 2 Mental Health Institute, the Second Xiangya Hospital of Central South University, Changsha, China; 3 Center for Statistical Science, Hong Kong University of Science & Technology, Clear Water Bay, Hong Kong, China; 4 The Second Affiliated Hospital of Jining Medical College, Jining, Shandong, China; 5 State Key Laboratory of Molecular Neuroscience, Hong Kong University of Science & Technology, Clear Water Bay, Hong Kong, China; Sudbury Regional Hospital, CANADA

## Abstract

Substance dependence is a frequently observed comorbid disorder in schizophrenia, but little is known about genetic factors possibly shared between the two psychotic disorders. *GABRB2*, a schizophrenia candidate gene coding for GABA_A_ receptor β_2_ subunit, is examined for possible association with heroin dependence in *Han* Chinese population. Four single nucleotide polymorphisms (SNPs) in *GABRB2*, namely rs6556547 (S1), rs1816071 (S3), rs18016072 (S5), and rs187269 (S29), previously associated with schizophrenia, were examined for their association with heroin dependence. Two additional SNPs, rs10051667 (S31) and rs967771 (S32), previously associated with alcohol dependence and bipolar disorder respectively, were also analyzed. The six SNPs were genotyped by direct sequencing of PCR amplicons of target regions for 564 heroin dependent individuals and 498 controls of Han Chinese origin. Interestingly, it was found that recombination between the haplotypes of all-derived-allele (H1; OR = 1.00) and all-ancestral-allele (H2; OR = 0.74) at S5-S29 junction generated two recombinants H3 (OR = 8.51) and H4 (OR = 5.58), both conferring high susceptibility to heroin dependence. Additional recombination between H2 and H3 haplotypes at S1-S3 junction resulted in a risk-conferring haplotype H5 (OR = 1.94x10^9^). In contrast, recombination between H1 and H2 haplotypes at S3-S5 junction rescued the risk-conferring effect of recombination at S5-S29 junction, giving rise to the protective haplotype H6 (OR = 0.68). Risk-conferring effects of S1-S3 and S5-S29 crossovers and protective effects of S3-S5 crossover were seen in both pure heroin dependent and multiple substance dependence subgroups. In conclusion, significant association was found with haplotypes of the S1-S29 segment in *GABRB2* for heroin dependence in Han Chinese population. Local recombination was an important determining factor for switching haplotypes between risk-conferring and protective statuses. The present study provide evidence for the schizophrenia candidate gene *GABRB2* to play a role in heroin dependence, but replication of these findings is required.

## Introduction

Substance dependence is a common comorbid disorder in schizophrenia with the highest abuse rate from alcohol (43.1%-65%), followed by cannabis (50.8%), nicotine (28.5%), and cocaine dependence (23%) [1]. Mechanisms involved in the comorbidity of schizophrenia and substance dependence are poorly understood, but much of the proposed models agree on the complex interaction between biological and genetic factors: (1) existence of common genetic risk factors in the dopamine (DA) pathway that leads to the concomitant onset of both disorders; (2) the self-medication hypothesis which states that substance abuse is used as a means to alleviate negative and deficit symptoms of schizophrenia [2].

Type A γ-aminobutyric acid (GABA_A_) receptor is the major inhibitory receptor in the central nervous system (CNS) and is responsible for triggering neuronal hyperpolarization through modulation of the influx of chloride ions [3]. The receptor is a target of CNS-activating compounds that include alcohol, benzodiazepines, barbiturates, and neurosteroids [4–6]. In mammalian brains, the most widely expressed hetero-metameric combinations of GABA_A_ receptor comprises of α1, β2, and γ2 subunits, which are encoded by the α1 subunit (*GABRA1*), β2 subunit (*GABRB2*), and ү2 subunit (*GABRG2*) genes [3]. Such a diverse localization of GABA_A_ β2 subunit in the mammalian brain raised the possibility on the possible overlap in the genetic susceptibility of schizophrenia and substance dependence. In case-control studies, significant association of *GABRB2* has been observed with various psychotic disorders including autism [7], bipolar disorder [8], epilepsy [9], schizophrenia [10–13], and alcohol dependence [14].

Heroin dependence is a complex brain disease with a severe impairment of neurological processes, preoccupation, and convulsive behavior upon withdrawal [15]. Intense euphoric effects from consuming a high dose of heroin are the major cause of its dependence [16], which is achieved through modification of gene expression and signal transduction [17]. Although precise mechanisms mediating pharmacological and genetic interaction of heroin with the GABA_A_ receptor are unknown, recent studies in rodents have shown that reinforcement of euphoric effects and self-administration of heroin are mediated by the hyperpolarization of GABAergic interneurons in the ventral tegmental area (VTA), thereby stimulating dopamine release in the nucleus accumbens (NAc) [18–20]. Moreover, significant association of *GABRB2* with opioid (heroin and cocaine) dependence has been detected in the European and African American populations [21], though significant association of this gene with heroin dependence alone has not been found in the Han Chinese population [22]. Although role of *GABRB2* in susceptibility to heroin dependence is unclear, twin studies have suggested that a common, heritable genetic factor confers a strong risk to substance dependence [23–24], while heroin dependence has been shown to possess a relatively high level of heritability (40–60%). Thus, these results suggest that a heritable genetic factor in *GABRB2* can be the common determinant of schizophrenia and heroin dependence [25].

In this study, we explored the role of intronic single nucleotide polymorphisms (SNPs) in *GABRB2* in susceptibility to heroin dependence. Based on the possibility of the shared genetic susceptibility to schizophrenia and substance dependence, four SNPs that have been reported for their significant association with schizophrenia in the Han Chinese, Japanese, German, and Portuguese populations have been selected [10,13]. These polymorphisms have also been previously reported for their positive selection of their derived alleles in schizophrenic patients [26]. In addition, two additional SNPs that have been reported to be positively associated with alcohol dependence [27] and bipolar disorder [8], respectively, have been included in this study. All of the six SNPs, however, have not been previously associated with heroin dependence. Accordingly, we examined allele, genotype, and haplotype frequencies of the selected SNPs in heroin dependent individuals and control groups in the Han Chinese population.

## Methods and Materials

### Ethics statement

A written informed consent was obtained from subjects prior to the study. Approval of the study was obtained from the ethical committees of Second Xiangya Hospital of Central South University and Beijing Normal University.

### Study subjects

Subjects with current or past diagnosis of heroin dependence were considered as the disease group. Seven hundred and thirty-six unrelated individuals (257 female patients; 477 male patients), diagnosed as heroin dependent according to the criteria in the Diagnostic and Statistical Manual and Mental Disorders fourth edition (DSV-IV) [28], were recruited from psychotic wards of the Second Xiangya Hospital of Central South University. A subset of subjects suffered from additional substance abuse including alcohol (ALC), sedative (SED), euphoriant (EUP), and hallucinogen (HAL). Patients with family history or suffering from other forms of psychotic disorders were excluded from the study. Information of each patient’s age, gender, and duration of heroin dependence were supplied by the doctors from the hospital. Unrelated healthy subjects without any personal or family history of substance dependence or psychotic disorders served as controls. Two groups of control subjects were recruited from Second Xiangya Hospital of Central South University (42 female subjects; 182 male subjects) and Beijing Normal University (176 female subjects; 150 male subjects), respectively. All subjects were from the Han Chinese population.

### Target region amplification by polymerase chain reaction (PCR)

Genomic DNA was extracted from peripheral blood samples by using DNA purification kit (American Biosciences Corp., Uppsala, Sweden). Four intronic regions in *GABRB2* extending from 160,898,693 bp to 160,755,432 bp in chromosome 5 were amplified (contig accession number: NT_023133.12; messenger RNA accession number: NM_021911): (1) 2.2 kbp region containing rs6556547 (S1), rs1816071 (S3), and rs1816072 (S5) [4.1 kbp upstream to 1.9 kbp upstream of 5’ end of Exon 9]; (2) 1.4 kbp region containing rs187269 (S29) [2.7 kbp downstream to 0.7 downstream of 5’ end Exon 9; (3) 0.7 kbp region containing rs10051667 (S31) [140.6 kbp upstream to 139.9 kbp upstream of 5’ end of Exon 9] (4) 0.8 kbp region containing rs967771 (S32) [4.7 kbp upstream to 3.5 kbp upstream of 5’ end of Exon 9]. Specific primers were designed by Primer-BLAST of the National Center for Biotechnology Information (http://www.ncbi.nlm.nih.gov/tools/primer-blast/) with avoid-low-complexity-region-for-primer-selection parameter. Primers with less than five hits in the human genome were chosen as forward and reverse primers. The primers used to amplify these fragments were shown in S1 Table.

Nested polymerase chain reaction (PCR) was employed to amplify the target regions. DNA fragment was amplified in a 20 μl reaction mixture containing 10 ng of genomic DNA, 150 nM of each primer, 200 μM of each deoxyribonucleotide triphosphate (dNTP), 1.5 mmol/L magnesium chloride (MgCl_2_), and 1 U *Taq* polymerase (Amersham Biosciences Corp., NJ). PCR amplification consisted of an initial denaturation at 95°C for 5 minutes, 30 cycles of 30 seconds at 95°C, 30 seconds at annealing temperature optimum for each primer pair, elongation at 72°C for the length of time adjusted to the size of the desired amplicon, and followed by a final elongation step at 72°C for 7 minutes. A small amount of PCR product was electrophoresed in a 0.7–2.0% agarose gel, stained with ethidium bromide, and was visualized under ultraviolet light (UV) to confirm successful amplification.

### SNP identification from direct sequencing of PCR products

Amplified PCR products were purified by ethanol. Absolute ethanol was added to the PCR products to a final concentration of 75% and the mixture was stored at -20°C overnight. The mixture was centrifuged at 3,000 rpm for 30 minutes and the resulting precipitate was washed in 70% ethanol for two additional rounds, which was then centrifuged at 3,000 rpm for 20 minutes. The precipitates were air-dried and dissolved in 1X Tris-Cl (Affymetrix, Inc., OH) and ethylenediaminetetraacetic acid (EDTA; Grand Island, NY) buffer. Each sequencing reaction was performed in a 15 μl reaction mixture containing ~100 ng purified PCR products, 480 nmol/L sequencing primer, and 0.75 μl BigDye^®^ Terrminator version 3.1 (Applied Biosystems, CA). Sequencing reaction consisted of an initial denaturation at 95°C for 1 minute, 30 cycles of 10 seconds at 95°C, 5 seconds at 50°C, and 4 minutes at 60°C. Products from the sequencing reaction were purified with ethanol using the same protocol. Purified products were then dissolved in 10 μl Hi-Deionized Formamide (Applied Biosystems), denatured at 95°C for 1 minute, and were sequenced using the ABI PRISM^®^ model 3100 capillary DNA sequencer (Applied Biosystems). All SNPs (S1, S3, S5, S29, S31, and S32) were genotyped by at least two independent researchers with the CODON CODE ALIGNER software, version 5.0.1 (http://www.codoncode.com/index.htm). Chromatogram reads with low-quality score were re-sequenced. Refer to S1 Dataset for information on gender, age, genotype, duration of heroin dependence, and status of multiple substance dependence of each patient and control individual.

### Linkage disequilibrium

Linkage disequilibrium (LD) was calculated using the Haploview software, version 4.2 [29]. Strength of LD were represented by the standardized LD coefficients *D’* and *r* ^*2*^ [30–31]. LD plots of pairwise SNPs were constructed for gender-combined control groups.

### Statistical analysis

Hardy-Weinberg equilibrium (HWE) exact test was performed for the disease and control samples using the GENEPOP software, version 4.2 [32–33]. Enumeration-of-alleles function was selected with Markov chain parameters in default settings. Quantitative trait analysis on duration of heroin dependence was tested with linear regression and linear-by-linear association test using the SPSS software, version 22 (SPSS Inc., Chicago, IL).

Disease association analysis was performed using the UNPHASED software, version 3.1.7 [34]. Overall association of SNPs with heroin dependence was computed by likelihood ratio statistics comparing allele, genotype, and haplotype frequencies. Effects of additional substance dependence (ALC, SED, EUP, and HAL) on relative susceptibility to heroin dependence were analyzed in haplotypes showing significant association with heroin dependence. *P*-value below 0.05 was considered as a statistically significant value for an association. All *P-*values showing significant associations were then reviewed by the Bonferroni correction (*P_Corr_*) and global permutation test (*P_Global_*). The global permutation test was based on the null hypothesis that all odds ratios of haplotypes were equal and *P*-value was corrected after 10,000 permutations. Moreover, the resampling method was employed as an additional means to correct the significance of an overall association of the SNPs, as reported in Lo *et al*., 2007 [13]. This method tested whether significant association with heroin dependence can be maintained when gender ratio and sample size of disease and control groups were equalized. A homemade R-script was used to create datasets with equal sample size and gender ratio and the sample size was based on the number of females in the control group (S1 Text). A total of 1,000 randomly extracted datasets without replacement were produced. Each dataset was tested for association with heroin dependence. Only datasets showing significant association with heroin dependence after the Bonferroni correction (*P_Corr_* < 0.05) were included in the results section unless otherwise indicated.

## Results

**Fig 1:**
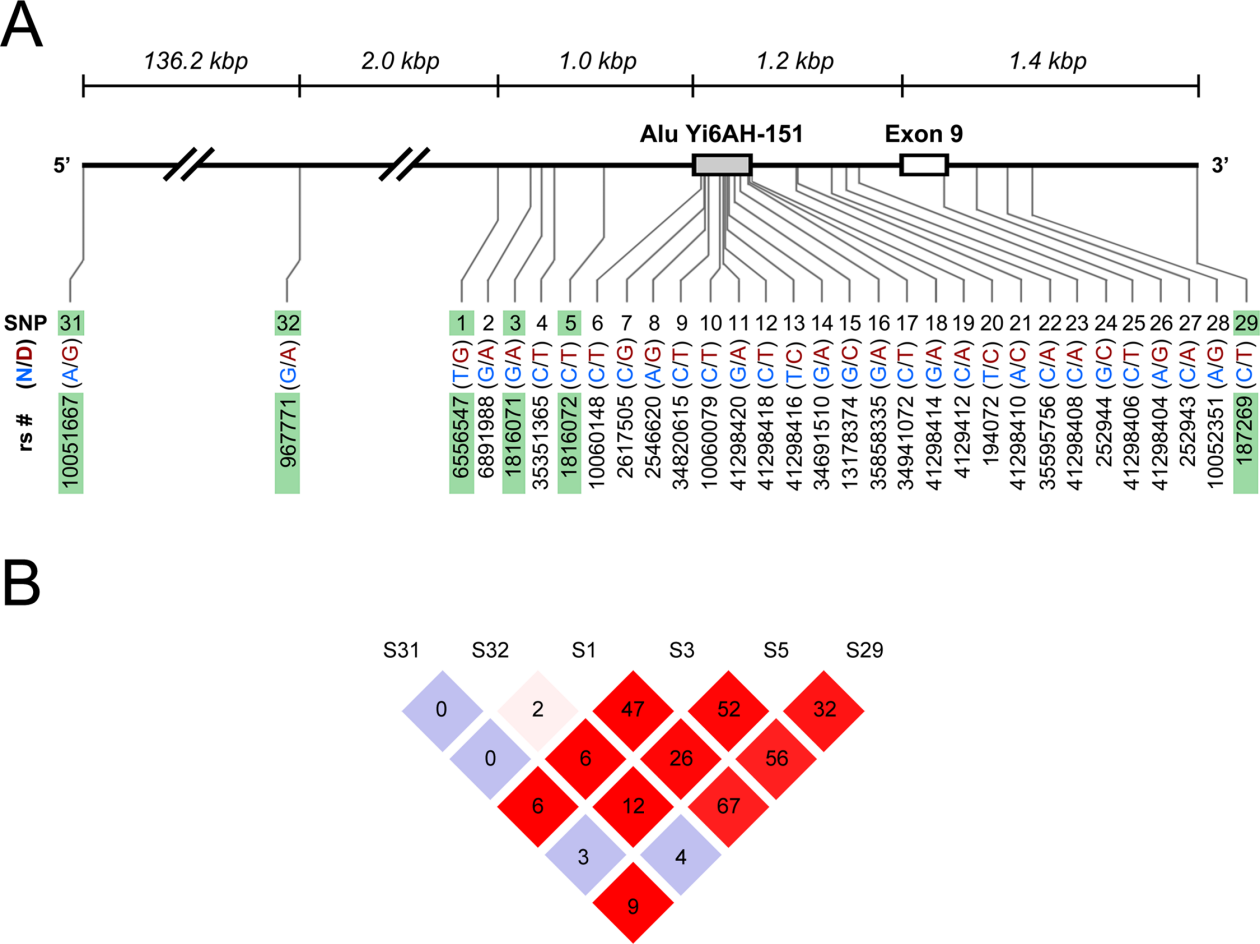
Schematic presentation of SNPs in *GABRB2*. (A) Positions of SNPs in *GABRB2*, with reference to contig NT_023133.12 and messenger RNA NM_021911. The region spans from 160,898,711 bp to 160,755,827 bp in chromosome 5. Solid black line represent introns. A flanking grey box indicates the human specific Alu insert Yi6AH-151 and a white box represents Exon 9. SNP label with ‘rs’ prefix is based on the Single Nucleotide Polymorphism database (dbSNP; www.ncbi.nih.gov/SNP). Ancestral and derived (N/D) alleles are provided for each SNP in blue and red, respectively. Six SNPs (S1, S3, S5, S29, S31, and S32) studied for heroin dependence are highlighted in green. (B) Linkage equilibrium plot of pairwise SNPs in *GABRB2* in control groups. *r* ^*2*^ value corresponding to each SNP pair is displayed in the square (*r* ^*2*^ x 100). Background color of squares represents the ratio of *D’* value to the log likelihood of odds ratio (LOD) corresponding to each SNP pair. Squares are colored in darker red if *D’* < 1.000 and LOD ≥ 2.00, white if *D’* < 1.000 and LOD < 2.0, and grey if *D’* = 1.000 and LOD < 2.0.

The four intronic SNPs in *GABRB2* previously reported for their significant association with schizophrenia and positive selection of their derived alleles [26, 35], namely rs6556547 (S1), rs1816071 (S3), rs1816072 (S5), and rs187269 (S29), were genotyped and analyzed in our study. Two additional SNPs rs10051667 (S31) and rs967771 (S32), reported for their positive association with alcohol dependence and bipolar disorder [8, 27], respectively, were also included in this study. Schematic location of these SNPs was shown in Fig 1A. With the exception of S31 and S32, specific naming adopted for each SNP in parenthesis followed the convention used in [26]. Missing genotype information at any SNP locus was not inferred in reference to other genetic markers.

### Subject selection

Patient and control samples with missing genotype information for S1, S3, S5, or S29 or missing gender information have been excluded in this study (S2 Table). After exclusion, there were 514 heroin dependent individuals (HER) (176 female patients; 338 male patients; mean age 32.9 ± 7.0), control samples recruited from Changsha (CON1) (34 female subjects; 147 male subjects; mean age 33.5 ± 6.3) and Beijing (CON2) (171 female subjects; 146 male subjects; mean age 28.2 ± 10.7). For S31 and S32, number of successfully genotyped samples in HER and CON1 groups was low due to the low quality of available primers used for template amplification and difficulty to amplify the target regions containing these markers. In addition, approximately 61.9% (318 subjects) of heroin dependent individuals had been diagnosed with additional forms of substance dependence, with the highest rate from sedative (16.7%; 86 subjects), followed by alcohol (15.0%; 77 subjects), euphoriant (3.1%; 16 subjects), and hallucinogen (1.8%; 9 subjects).

### Association analysis of individual SNPs for heroin dependence

In order to equalize the sample size of the heroin dependent and control groups and exclude differences in geographical origins of subject recruitment, allelic and genotypic comparisons were made between CON1 and CON2 groups (S3 Table). Significant association was not detected in allele or genotype level at any SNP locus (*P* > 0.05) and significant deviation from HWE (*P* > 0.05) was not observed in neither control groups. Thus, the two control groups were combined as one group (CON) in the subsequent statistical analyses.

With the exception of S29, all genetic markers did not significantly deviate from HWE (*P* > 0.05) in HER and CON groups (S4 Table). Allelic and genotypic associations of these markers with heroin dependence (S4 Table) were also not significant (*P* > 0.05). In addition, LD plot of pairwise SNPs in gender-combined combined controls (CON) showed that the four SNPs, S1, S3, S5, and S29, are moderately linked (*D’* ≥ 0.929, *r* ^*2*^ ≥ 0.264), while LD with S31 or S32 were not linked despite high *D’* values (D’ = 1.000, *r* ^*2*^ ≤ 0.122) (Fig 1B; S4 Table). Moreover, quantitative analysis on duration of heroin dependence revealed that individual’s genotype was not associated with duration of heroin dependence, regardless of gender (S5 Table).

### Haplotype association analysis for heroin dependence

All haplotypes showing significant association with heroin dependence after the Bonferroni correction and global permutation test contained S29 (Table 1). The strongest association was found in S1-S5-S29 (*P*_*Corr*_ = 8.637x10^-13^, *P*_*Global*_ = 0.0001). S1-S3-S5-S29, S1-S3-S29, and S1-S29 were the next strongly associated haplotypes by this order (S1-S3-S5-S29 haplotype *P*_*Corr*_ = 4.150x10^-10^, *P*_*Global*_ = 0.0001; S1-S3-S29 haplotype *P*_*Corr*_ = 4.624x10^-10^, *P*_*Global*_ = 0.0001; S1- S29 haplotype *P*_*Corr*_ = 2.109x10^-9^, *P*_*Global*_ = 0.0001). Interestingly, only haplotypes that contain SNPs in the S1-S29 segment (S1, S3, S5, or S29) but not those that contain S31 and/or S32 were significantly associated in all 1,000 resampled datasets (*P* < 0.05).

**Table 1:** Overall haplotype association analysis for heroin dependence.

*Haplotype* ^*a*^						*χ* ^*2*^	*P*	*P*_*Corr*_	*P*_*Global*_	*Number of Significant Resampled Datasets*
*S31*	*S32*	*S1*	*S3*	*S5*	*S29*					
		**X**			**X**	**45.561**	**7.031x10** ^**-10**^	**2.109x10** ^**-9**^	**0.0001**	**1000**
			**X**		**X**	**36.083**	**7.191x10** ^**-8**^	**2.157x10** ^**-7**^	**0.0001**	**1000**
				**X**	**X**	**37.724**	**1.986x10** ^**-8**^	**5.958x10** ^**-8**^	**0.0001**	**1000**
X			X		X	21.919	0.001	0.008	0.0018	454
	X		X		X	27.263	5.070x10^-5^	2.535x10^-4^	0.0002	294
	X			X	X	20.033	0.001	0.006	0.0012	312
		**X**	**X**		**X**	**61.796**	**6.605x10** ^**-11**^	**4.624x10** ^**-10**^	**0.0001**	**1000**
		**X**		**X**	**X**	**75.322**	**1.234x10** ^**-13**^	**8.637x10** ^**-13**^	**0.0001**	**1000**
			**X**	**X**	**X**	**50.155**	**1.347x10** ^**-8**^	**9.427x10** ^**-8**^	**0.0001**	**1000**
X	X	X			X	27.062	0.004	0.049	0.0023	94
X	X		X		X	34.594	7.025x10^-5^	6.323x10^-4^	0.0001	426
X	X			X	X	24.080	0.004	0.038	0.0030	129
X		X	X		X	31.033	0.001	0.012	0.0024	199
X		X		X	X	32.453	0.001	0.014	0.0014	129
X			X	X	X	29.209	0.001	0.012	0.0014	308
	X	X	X		X	36.366	7.281x10^-5^	7.281x10^-4^	0.0002	294
	X	X		X	X	32.411	1.690x10^-4^	1.521x10^-3^	0.0003	312
	X		X	X	X	33.725	9.979x10^-5^	8.981x10^-4^	0.0002	503
		**X**	**X**	**X**	**X**	**77.541**	**3.193x10** ^**-11**^	**4.150x10** ^**-10**^	**0.0001**	**1000**
X	X	X	X		X	45.422	6.566x10^-5^	9.849x10^-4^	0.0003	339
X	X	X		X	X	36.470	8.869x10^-4^	0.012	0.0008	229
X	X		X	X	X	37.443	1.893x10^-4^	2.272x10^-3^	0.0002	335
X		X	X	X	X	39.544	0.002	0.043	0.0037	104
	X	X	X	X	X	40.872	5.800x10^-4^	0.009	0.0005	247
X	X	X	X	X	X	46.024	2.943x10^-4^	0.005	0.0005	246

Overall haplotype association analysis for heroin dependence comparing the heroin dependent group (HER) with the combined control (CON) group. Each SNP included in a haplotype association test is denoted by ‘X’. *P*-value computed by the likelihood ratio test is the average of all possible haplotypes of an association and this was corrected by the Bonferroni method (*P_Corr_*) and global permutation test (*P*_*Global*_ *)*. Number of resampled datasets showing significant associations after the Bonferroni correction was counted (*P*_*Corr*_ < 0.05). Only haplotypes maintaining significant association with heroin dependence after the Bonferroni correction and global permutation test are shown in the table.

^a^ Haplotypes that only contain SNPs in the S1-S29 segment (S1, S3, S5, and S29) are shown in bold font.

**Fig 2:**
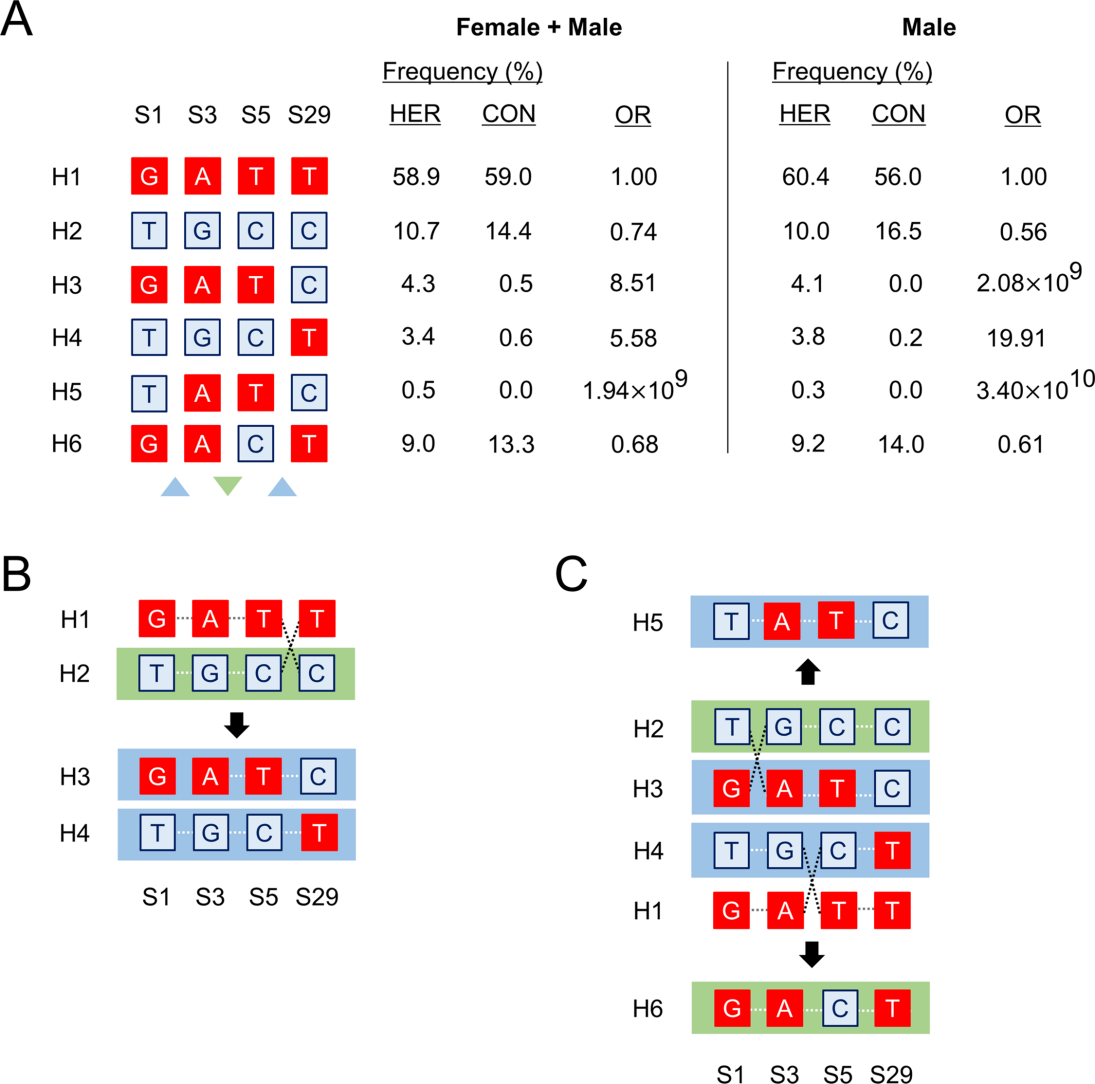
Recombination in haplotypes in association with heroin dependence. Individual 4-SNPs haplotypes (H2-H6), containing S1, S3, S5, and S29, significantly associated with (*P* < 0.05; *P*_*Global*_ < 0.05) with heroin dependence are separately analyzed in male + female (case n = 564; control n = 498) and male samples (case n = 338; control n = 293). A blue square block represents the ancestral allele and a red square block represents the derived allele. Refer to S6 Table for further information on this figure. (A) Haplotype frequencies in female + male and male heroin dependent (HER) and combined control (CON) groups. Odds ratios (OR) are based on the all-derived-allele haplotype (H1). Odd ratio of 1.00 serves as the baseline for determining haplotype risks. An upward arrowhead in blue color shows risk-conferring effect of recombination and a downward arrowhead in green color shows a protective effect of recombination. (B) Effect of single recombination at S5-S29 junction on haplotype risks; and (C) Effect of additional recombination at S1-S3 and S3-S5 junctions on haplotype risks. (B-C) Dashed black intersecting lines represent major crossover points. Haplotype in blue background represents a risk-conferring haplotype (OR > 1.00) and haplotype in green background represents a protective haplotype (OR < 1.00).

Given the extremely low p-values of haplotypes, containing S1, S5, S5, and S29, associated with heroin dependence, role of haplotype level recombination in susceptibility to heroin dependence was further examined. Exhaustive analysis of all possible four-SNPs haplotypes was performed to observe the combined effect of the four genetic loci in female + male and male samples. However, minimum haplotype frequency threshold was not used in this analysis to visualize active recombination of common and rare haplotypes. Homozygous derived allele haplotype (H1 haplotype) was included as a reference (Fig 2A). While there was no significant difference in frequency of H1 haplotype between HER and CON groups (*P* > 0.05), all-ancestral-allele haplotype (H2) showed significant association with heroin dependence (Female + male sample, OR = 0.74, 95% CI = 0.57–0.97, *P*_*Global*_ = 0.0183; Male sample, OR = 0.56, 95% CI = 0.40–0.79, *P*_*Global*_ = 0.0010) (Fig 2A). Moreover, additional analysis of haplotypes showed that odds ratio was flipped by multiple recombination, which was observed in both female + male and male samples. Single recombination between H1 and H2 haplotypes at S5-S29 junction (Fig 2B) resulted in a constitutive increase of susceptibility to heroin dependence and removed the protective nature of H2 haplotype, which were observed in H3 and H4 haplotypes (Female + male sample, H3 haplotype OR = 8.51, 95% CI = 3.04–23.83, *P*_*Global*_ = 0.0001; H4 haplotype OR = 5.58, 95% CI = 2.28–13.64, *P*_*Global*_ = 0.0001; Male sample, H3 haplotype OR = 2.08x10^9^, 95% CI = 1.73x10^9^-2.54x10^9^, *P*_*Global*_ = 0.0001; H4 haplotype OR = 19.91, 95% CI = 2.69–147.30, *P*_*Global*_ = 0.0001). Additional recombination between H2 and H3 haplotypes at S1-S3 junction (Fig 2C) exacerbated the risk to heroin dependence, which was observed in H5 haplotype. This rare haplotype, occurring in frequency less than 1 percent in both HER and CON groups, was significantly associated in female + male sample, but not in male sample alone (Female + male sample, OR = 1.94x10^9^, 95% CI = 1.19x10^9^-3.18x10^9^, *P*_*Global*_ = 0.0460; Male sample, OR = 3.40x10^10^, 95% CI = 1.70x10^10^-6.82x10^10^, *P* > 0.05). Interestingly, additional recombination between H1 and H4 haplotypes at S3-S5 junction created H6 haplotype, which restored the protection from heroin dependence (Female + male sample, OR = 0.68, 95% CI = 0.51–0.91, *P*_*Global*_ = 0.0026; Male sample, OR = 0.61, 95% CI = 0.43–0.87, *P*_*Global*_ = 0.0049).

To confirm whether the flipping effect of recombination on the susceptibility to heroin dependence observed in the four-SNPs haplotypes was independent of the effect of other two markers, six-SNPs containing all markers employed in this study were analyzed. Consistent with previous findings, H2-H6 haplotypes showed the same trend in the susceptibility to heroin dependence, though only H3 and H4 haplotypes were significantly associated with heroin dependence in both female + male and male samples (S1 Fig). In addition, recombination at S1-S3 junction and S5-S29 junction had a negative effect on the susceptibility to heroin dependence, while recombination at S3-S5 junction restored the protection from heroin dependence (S1 Fig). Additional recombination at S31-S32 junction resulting in H7 haplotype seemed to exacerbate the risk to heroin dependence, but did not seem to significantly affect the risk conferred by recombination of other four SNPs (S1, S3, S5, and S29), while risk-conferring recombination at S1-S3 and S5-S29 junctions have been observed (S1 Fig). Taken together, these results suggest that S31 and S32 may not largely contribute to the haplotype recombination of S1, S3, S5, and S29 in susceptibility to heroin dependence. Thus, haplotypes containing S31 and/or S32 were excluded from further analyses relating to susceptibility to heroin dependence.

### Haplotype association analysis in multiple substance abuse subgroups

**Fig 3:**
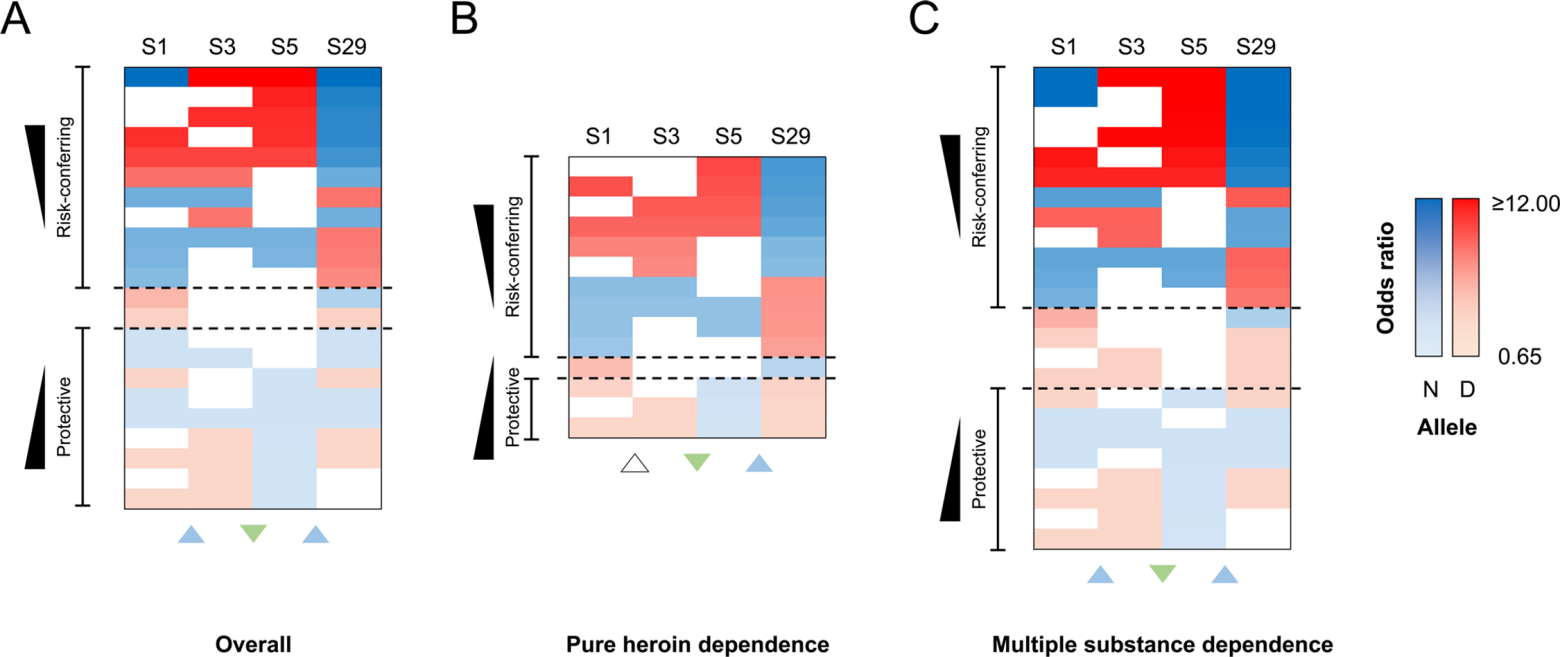
Odds ratio in multiple SNP-containing haplotypes in association with heroin dependence. Individual 2-, 3-, and 4-SNPs haplotypes, containing S1, S3, S5, and S29, significantly associated with heroin dependence (P < 0.05; P_*Global*_ < 0.05) are organized by their odds ratio (OR) in a descending order. Odd ratio of 1.00 serves as the baseline for determining haplotype risks. A blue square block represents the ancestral allele and a red square block represents the derived allele. An upward arrowhead in blue color shows risk-conferring effect of recombination and a downward arrowhead in green color shows protective effect of recombination. Neutral effect of recombination is represented by an upward arrowhead in white color. Dashed lines represent the cutoffs. Refer to S8 Table for further information on this figure. (A) Odds ratio of haplotypes in comparing overall heroin dependent (HER) and combined control (CON) groups (case n = 564; control n = 498). Odds ratio ≥ 4.73 is chosen as a cutoff for risk-conferring haplotypes to heroin dependence. Odds ratio ≤ 0.81 is chosen as a cutoff for protective haplotypes from heroin dependence. (B) Odds ratio of haplotypes in comparing pure heroin dependent subgroup and CON group (case n = 318; control n = 498). Odds ratio ≥ 3.55 is chosen as a cutoff for risk-conferring haplotypes to heroin dependence and OR ≤ 0.79 is chosen as a cutoff for protective haplotypes from heroin dependence. (C) Odds ratio of haplotypes in comparing multiple substance dependent subgroup and CON group (case n = 246; control n = 498). Odds ratio ≥ 5.72 is chosen as a cutoff for risk-conferring haplotypes to heroin dependence. Odds ratio ≤ 0.78 is chosen as a cutoff for protective haplotypes from heroin dependence.

Effects of multiple substance dependence in susceptibility to heroin dependence were analyzed in individual 2-, 3-, and 4-SNPs haplotypes containing only S1, S3, S5, and S29. In overall heroin dependent individual (HER) group (Fig 3A), risk-conferring haplotypes to heroin dependence (OR ≥ 4.78) showed recombination at S1-S3 junction and/or S5-S29 junction, while protective haplotypes (OR ≤ 0.81) showed double recombination at S3-S5 and S5-S29 junctions. In pure heroin dependent individual subgroup (Fig 3B), risk-conferring haplotypes (OR ≥ 3.55) to heroin dependence only showed recombination at S5-S29 junction, while all of the protective haplotypes (OR ≤ 0.79) exhibited double recombination at S3-S5 and S5-S29 junctions. Interestingly, recombination at S1-S3 junction was not observed. In multiple substance dependent individual subgroup (Fig 3C), risk-conferring haplotypes to heroin dependence (OR ≥ 5.72) showed recombination at S1-S3 junction and/or at S5-S29 junction, while protective haplotypes (OR ≤ 0.78) showed double recombination at S3-S5 and S5-S29 junctions. Furthermore, number of protective haplotypes was observed in multiple substance dependence subgroup (8 haplotypes) was higher than pure heroin dependence (3 haplotypes) subgroup, while similar number of risk-conferring haplotypes was seen in both subgroups (Multiple substance dependence subgroup, 12 haplotypes; pure heroin dependence subgroup, 10 haplotypes) (Fig 3B and 3C).

## Discussion

We examined the role of in *GABRB2* in susceptibility to heroin dependence in the Han Chinese population. Although we failed to show that genetic markers in *GABRB2* (S1, S3, S5, S29, S31, and S32) were associated with heroin dependence in allele and genotype levels (*P* > 0.05), very significant associations were detected at haplotype level, especially in haplotypes only containing SNPs in the S1-S29 segment (S1, S3, S5, and S29) (*P*_*Corr*_ < 0.001; *P*_*Global*_ = 0.0001) (Table 1). In addition, LD plot of pairwise SNPs in CON group also showed that only S1, S3, S5, and S29 but not S31 and S32 were moderately linked (Fig 1B), indicating that S31 and S32 may not play a significant role in susceptibility to heroin dependence. Taken together, these results suggested the strong link of the S1-S29 segment in *GABRB2* with susceptibility to heroin dependence.

Results of haplotype level associations support the existence of common genetic predisposition to schizophrenia and heroin dependence. Genetic markers employed in this experiment have been replicated in studies with different populations with schizophrenia [10,13]. In particular, inclusion of S29 in all significantly associated haplotypes (Table 1) suggested that S29 might be an important genetic marker determining the risk of haplotype blocks. Compared to the initial report on S29 ancestral allele (OR = 1.93) by Lo WS *et al*., 2004 [10], and (OR = 1.65) by Zhao X. *et al*., 2007 [12] in case-control studies conducted with schizophrenic patients, S29 allele in this report (S4 Table) conferred a weaker risk to heroin dependence (OR = 1.14). In haplotype level, significant associations of S3-S29 and S5-S29 allele haplotypes with bipolar disorder and schizophrenia were reported in the Han Chinese and German populations [36]. In both populations, frequency of S3-S29 and S5-S29 D-D allele haplotypes was higher in patients with bipolar disorder and schizophrenia than control groups. However, we observed that S3-S29 D-N and S5-S29 D-N allele haplotypes were significantly associated in heroin dependence and both haplotypes occurred more frequently in heroin dependent individuals than in controls (S8 Table). Although allele combinations of S3-S29 and S5-S29 haplotypes associated with bipolar disorder and schizophrenia were different from those associated with heroin dependence, these observations might be due to complex genetic basis involved in these disorders or due to the ‘flip-flop’ phenomenon from multilocus effects and interlocus correlations [37].

Susceptibility to heroin dependence was closely linked to local recombination events. Co-occurrence of recombination hotspot and positive selection of alleles in the S1-S29 segment in *GABRB2* was reported in our previous findings [26, 35], which are mediated by the insertion of Alu repeats [38]. In this study, we analyzed all 4-SNPs haplotypes consisting of S1, S3, S5, and S29 regardless of their frequency to observe active recombination events of common and rare haplotypes. Both common (with allele frequency > 1%) and rare variants (with allele frequency < 1%) have been shown to play a role in genetic susceptibility to common and complex diseases [39–41]. Consistent with these results, all significantly associated haplotypes with heroin dependence in HER group were commonly observed with frequency above 1 percent, while H5 haplotype was rarely observed with frequency below 1 percent (Fig 2A). We also found that local recombination or gene conversion played a critical role in determining the odds for an individual’s predisposition to heroin dependence. Local recombination at S1-S3 junction and at S5-S29 junction conferred increased the risk to heroin dependence, while subsequent gene conversion at S3-S5 junction restored the protection from heroin dependence (Fig 2B and 2C). Moreover, this study included S31 and S32 which have been shown to be positively associated with alcohol dependence and bipolar disorder, respectively. Including these two markers in haplotype analysis showed the same trend of risk-conferring and protective nature of local recombination observed in the S1-S29 segment (S1 Fig), but this inclusion also weakened the association of haplotypes containing S1, S3, S5, and S29 with heroin dependence (Table 1). Since the distance from S31 and S32 to S1 is more than 2.0 kbp (Fig 1), it is likely that S31 and S32 may not affect the gene conversion observed in the haplotype block containing S1, S3, S5, and S29.

Complex interaction of multiple substance abuse with recombination was further analyzed in 2-, 3-, and 4-SNPs haplotypes. Detrimental effects of gene conversion at S1-S3 and S5-S29 junctions, and protective effects of gene conversion at S3-S5 junction were observed in overall heroin dependent group (HER) as well as in both pure heroin dependence and multiple substance dependence subgroups (Fig 3). Interestingly, number of protective haplotypes from heroin dependence was higher in multiple substance than pure heroin dependence subgroups (Fig 3B and 3C). This evidence supports the self-mediation hypothesis in schizophrenic patients [2], by which increase in the number of protective haplotypes may have helped to reduce the psychotic symptoms or the effects of anti-psychotic drugs for schizophrenia.

Comparison of haplotype level associations in heroin dependence suggested that genetic studies focusing on the disease associations in allele and genotype levels could result in incomplete conclusions. Protective odds ratio of the ancestral S1 and S5 alleles (S4 Table) was not consistent to the results at haplotype level. While inclusion of the ancestral S5 allele was essential in providing protection to heroin dependence, as observed in H2 and H6 haplotypes, inclusion of the ancestral S1 allele did not seem to be involved in this protection (Fig 2A). Based on these findings, we stress the importance of haplotype level analysis in case-control association studies. In association studies focusing on a small number of polymorphisms, detection of significant disease associations in allele and genotype level is often difficult due to the complex, multigenetic nature of psychotic disorders, random variation, or inappropriate correction for multiple testing [42–43]. In particular, formation and disruption of haplotype blocks in the disease group are increasingly believed to be an important guideline in detecting susceptibility to psychotic disorders, occurring extensively in recombination hotspots [44–45].

The four SNPs, S1, S3, S5, and S29, localized in the intronic regions of *GABRB2* have been reported to have a regulatory function of GABA_A_ receptor activity. In particular, the derived alleles of these markers were found to increase the mRNA expression of the total β2 subunit of the GABA_A_ receptor [26]. As the majority of the GABA_A_ receptor consists of the β2 subunit [3], the derived alleles of the four SNPs would enhance the inhibitory properties of the receptor. Based on these findings, all-ancestral-allele four-SNPs haplotype (H2) (Fig 2A) would reduce neuronal hyperpolarization in the CNS, thereby providing protection from recurrent and self-administrative effects of heroin dependence [18–20]. Interestingly, derived alleles of the four SNPs were observed more frequently in schizophrenic patients than controls [26], indicating that positive selection of the four SNPs plays an important role in determining susceptibility to schizophrenia and heroin dependence. In this light, risk-conferring and protective effects of gene conversion observed within the four SNPs suggests potential significance of simultaneous recombination towards susceptibility to heroin dependence.

Accordingly, the present study suggested the shared genetic basis of *GABRB2* in schizophrenia and heroin dependence. Considering the uniqueness of human recombination hotspots and positive selection of the alleles in *GABRB2* [26,35], gene conversion within the intronic S1-S29 segment could be a potential cause leading to the pathogenesis of schizophrenia and heroin dependence. In this study, relatively large number of case (n = 564) and control (n = 498) samples was employed to study the association of six candidate SNPs with heroin dependence. Since studying 1 SNP and 500,000 SNPs with 80% power require 248 and 1,206 cases respectively [46], it can be regarded that the sample size used to detect significant associations in this study was large enough to achieve adequate power. Moreover, as significant disease associations are not easily detected in allele or genotype level, haplotype-level analysis would therefore be a critical step in understanding the genetic susceptibility to schizophrenia and heroin dependence.

## Supporting Information

S1 Dataset Demographic and genotype information of individuals employed in the study. (XLSX)

S1 Fig Recombination in six-SNPs haplotypes in association with heroin dependence. (DOCX)

S1 Table Sequences of primer pairs. (DOCX)

S2 Table Heroin dependent individuals and control samples from Changsha and Beijing. (DOCX)

S3 Table Allele and genotype association analysis between control samples from Changsha and Beijing. (DOCX)

S4 Table Association and linkage disequilibrium analysis of individual SNPs. (DOCX)

S5 Table Quantitative trait analysis on duration of heroin dependence. (DOCX)

S6 Table Four-SNPs haplotype association analysis for heroin dependence. (DOCX)

S7 Table Six-SNPs haplotype association analysis for heroin dependence. (DOCX)

S8 Table Multiple-SNPs haplotype association analysis for heroin dependence in substance dependence subgroups. (DOCX)

S1 Text R-script for samples resampling. (DOCX)
