# Supplementary material for: GABRB2 Haplotype Association with Heroin Dependence in Chinese Population
Source: PLoS One. 2015 Nov 12;10(11):e0142049. doi: 10.1371/journal.pone.0142049 (PMC4643001; doi:10.1371/journal.pone.0142049)
Supplement: S1 Fig — (DOCX) [file pone.0142049.s002.docx]

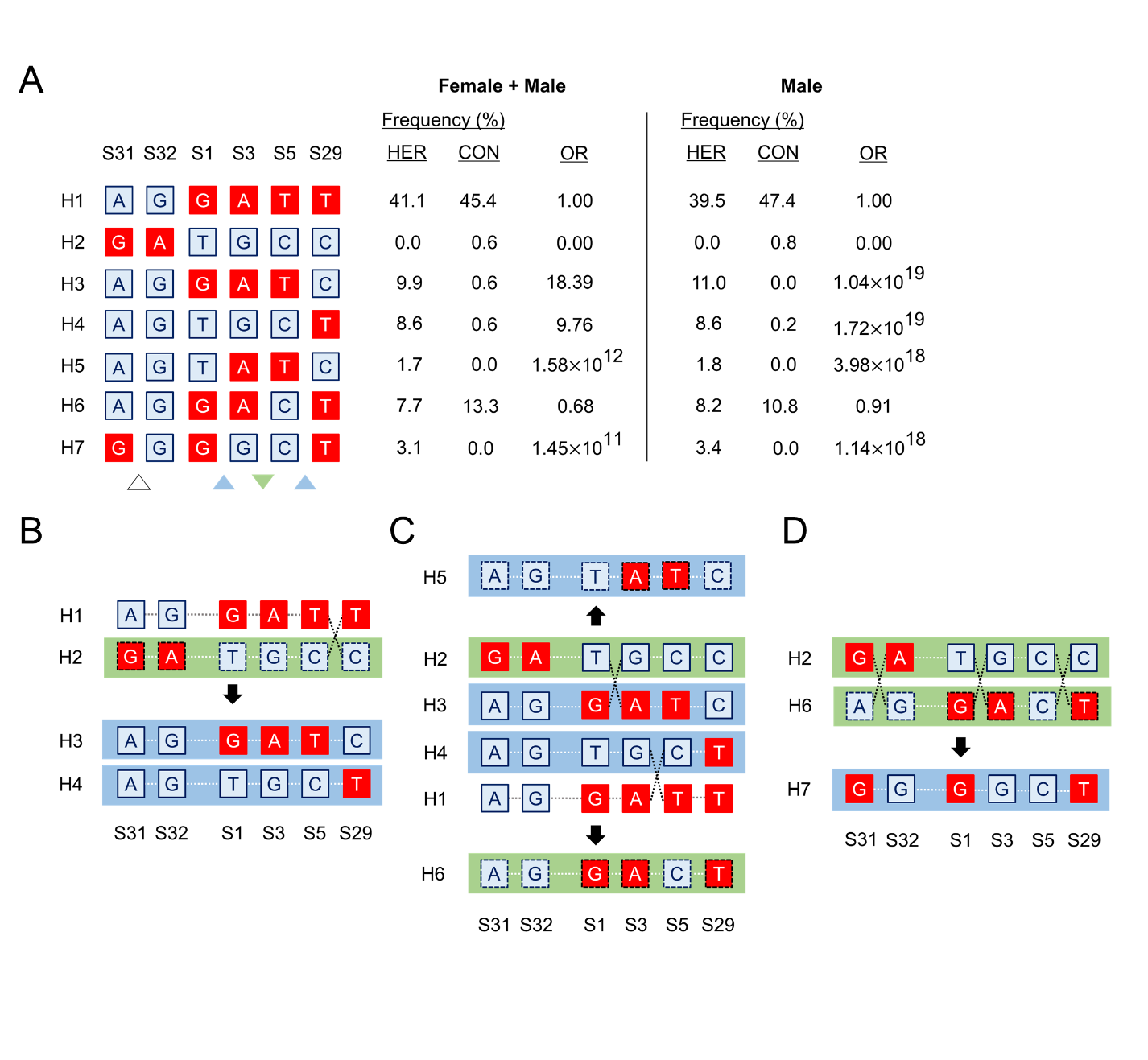


**S1 Fig.** Recombination in six-SNPs haplotypes in association with heroin dependence. Individual six-SNPs haplotypes (H2-H7) containing S1, S3, S5, S29, S31, and S32 are separately analysed in male + female (case n = 564; control n = 498) and male samples (case n = 338; control n = 293). A blue square block represents the ancestral allele and a red square block represents the derived allele. Refer to S7 Table for further information on this figure.

(A) Haplotype frequencies in female + male and male heroin dependent (HER) and the combined control (CON) groups. Odds ratios (OR) are based on the major allele haplotype (H1) consisting of the ancestral alleles for S31 and S32 and the derived alleles for S1, S3, S5, and S29. Odds ratio of 1.00 serves as the baseline for determining haplotype risks. An upward arrowhead in blue color shows risk-conferring effect of recombination and a downward arrowhead in green color shows a protective effect of recombination. Neutral effect of recombination is represented by an upward arrowhead in white color.

(B) Effect of single recombination at S5-S29 junction on haplotype risks; (C) Effect of additional recombination at S1-S3 and S3-S5 junctions on haplotype risks; and (D) Effect of additional recombination at S31-S32, S1-S3, and S5-S29 junctions on haplotype risks. (B-D) Haplotype information is based on female + male samples. Dashed black intersecting lines represent major crossover points. Haplotype in blue background represents a risk-conferring haplotype (OR > 1.00) and haplotype in green background represents a protective haplotype (OR < 1.00). Haplotype blocks outlined in black dash lines indicate those that are not significantly associated with heroin dependence (*P* > 0.05).
