# Supplementary material for: GABRB2 Haplotype Association with Heroin Dependence in Chinese Population
Source: PLoS One. 2015 Nov 12;10(11):e0142049. doi: 10.1371/journal.pone.0142049 (PMC4643001; doi:10.1371/journal.pone.0142049)
Supplement: S1 Table — (DOCX) [file pone.0142049.s003.docx]

**S1 Table.** Sequences of primer pairs.

| *DNA fragment^a^* | *Orientation* | *Name* | *Chromosomal position (bp)* | | *Sequence (5’-3’)* |
| --- | --- | --- | --- | --- | --- |
|  |  |  | *5’* | *3’* |  |
| Fragment 1 |  |  |  |  |  |
| For first PCR | Forward | 214F | 160,760,030 | 160,760,053 | ATGGAGGAAAGGTCCATATCTAGT |
|  | Reverse | R3N | 160,762,233 | 160,762,253 | CAACTGTCCTCACAATGACC |
| For second PCR | Forward | F2 | 160,760,078 | 160,760,097 | GGGAATGGTGCTCAGTAAAC |
|  | Reverse | R2 | 160,760,781 | 160,760,801 | GGACCTTGGACTTTCAGTGC |
| For sequencing | Forward | F2 | 160,760,078 | 160,760,097 | GGGAATGGTGCTCAGTAAAC |
|  |  |  |  |  |  |
| Fragment 2 |  |  |  |  |  |
| For first PCR | Forward | 217F | 160,755,432 | 160,655,459 | GAATGCTAGCAATACAGGTTGATGAC |
|  | Reverse | 218R | 160,757,389 | 160,757,412 | CCAGAGGACATTAGGCATTGTCTG |
| For second PCR | Forward | 218F | 160,756,341 | 160,756,359 | AGCACTTGCTGCACTAA |
|  | Reverse | 217R | 160,657,787 | 160,756,810 | ATTTTAAGTATGGCCCTTGGAAAC |
| For sequencing | Forward | 218F | 160,756,341 | 160,756,359 | AGCACTTGCTGCACTAA |
|  |  |  |  |  |  |
| Fragment 3 |  |  |  |  |  |
| For first PCR | Forward | 100F | 160,898,048 | 160,898,069 | ACTGGCATTTGAGAGCTAGG |
|  | Reverse | 100R | 160,898,693 | 160,898,712 | TCCAGCCCTATAACTGCCTA |
| For second PCR | Forward | 101F | 160,898,135 | 160,898,156 | ATAGCTGCTCCAGTTCTACC |
|  | Reverse | 101R | 160,898,654 | 160,898,673 | AGGTGATAACGGGACTGGTT |
| For sequencing | Reverse | 101R | 160,898,654 | 160,898,673 | AGGTGATAACGGGACTGGTT |
|  |  |  |  |  |  |
| Fragment 4 |  |  |  |  |  |
| For first PCR | Forward | 967F | 160,761,643 | 160,761,664 | TGAGAGGTTCAGTTGCGTCA |
|  | Reverse | 967R | 160,762,849 | 160,762,868 | GCCTGAAGTGGTTTGTAGGC |
| For second PCR | Forward | 968F | 160,762,049 | 160,762,070 | GTCTGAGAGCCCTGAGTTTC |
|  | Reverse | R1 | 160,762,431 | 160,762,455 | TGGAGTTCTAAAAGTATACCTTATC |
| For sequencing | Forward | 968F | 160,762,049 | 160,762,070 | TGGAGTTCTAAAAGTATACCTTATC |
|  |  |  |  |  |  |

^a^ Fragment 1 contains rs6556547 (S1), rs1816071 (S3), and rs1816072 (S5); Fragment 2 contains rs187269 (S29); Fragment 3 contains rs10051667 (S31); and Fragment 4 contains rs967771 (S32).
