# Supplementary material for: GABRB2 Haplotype Association with Heroin Dependence in Chinese Population
Source: PLoS One. 2015 Nov 12;10(11):e0142049. doi: 10.1371/journal.pone.0142049 (PMC4643001; doi:10.1371/journal.pone.0142049)
Supplement: S2 Table — (DOCX) [file pone.0142049.s004.docx]

**S2 Table.** Heroin dependent individuals and controls from Changsha and Beijing

| *Cohort* | *n* | | | | | |  | *Sex (female/male)* | *Age (years)* | *Duration of heroin dependence (years)* |  | *Substance abuse subgroups (n)* | | | | |
| --- | --- | --- | --- | --- | --- | --- | --- | --- | --- | --- | --- | --- | --- | --- | --- | --- |
|  | *S31* | *S32* | *S1* | *S3* | *S5* | *S29* |  |  |  |  |  | *Multiple* | *ALC* | *SED* | *EUP* | *HAL* |
| HER | 384 | 122 | 564 | 564 | 564 | 564 |  | 176/338 | 32.9 ± 7.0 | 10.0 ± 4.7 |  | 318 | 77 | 86 | 16 | 9 |
| CON1 | 143 | 100 | 181 | 181 | 181 | 181 |  | 34/147 | 33.5 ± 6.3 | ─ |  | ─ | ─ | ─ | ─ | ─ |
| CON2 | 0 | 0 | 317 | 317 | 317 | 317 |  | 171/146 | 28.2 ± 10.7 | ─ |  | ─ | ─ | ─ | ─ | ─ |

Demographic characteristics of heroin dependent group (HER) and control groups from Changsha (CON1) and Beijing (CON2) are shown. *n* refers to the number of successfully genotyped samples. Age and duration of heroin dependence are shown as mean ± standard deviation. Total number of heroin dependent individuals suffering from multiple substance abuse (Multiple) are shown. Multiple substances include alcohol (ALC), sedative (SED), euphoriant (EUP), and hallucinogen (HAL).
