# Supplementary material for: GABRB2 Haplotype Association with Heroin Dependence in Chinese Population
Source: PLoS One. 2015 Nov 12;10(11):e0142049. doi: 10.1371/journal.pone.0142049 (PMC4643001; doi:10.1371/journal.pone.0142049)
Supplement: S3 Table — (DOCX) [file pone.0142049.s005.docx]

**S3 Table.** Allele and genotype association analysis of control samples from Changsha and Beijing

| *SNP* | *D/N* |  | *Frequency* | | | | | | | | | | | |  | *HWE* | |  | *Allele* | |  | *Genotype* | |
| --- | --- | --- | --- | --- | --- | --- | --- | --- | --- | --- | --- | --- | --- | --- | --- | --- | --- | --- | --- | --- | --- | --- | --- |
|  |  |  | *D* | | | | *D/N* | | | | *N/N* | | | |  |  |  |  |  |  |  |  |  |
|  |  |  | *CON1* | | *CON2* | | *CON1* | | *CON2* | | *CON1* | | *CON2* | |  | *CON1* | *CON2* |  | *χ^2^* | *P* |  | *χ^2^* | *P* |
|  |  |  | *n* | *%* | *n* | *%* | *n* | *%* | *n* | *%* | *n* | *%* | *n* | *%* |  |  |  |  |  |  |  |  |  |
| S1 | G/T |  | 308 | 85.1 | 535 | 84.4 | 46 | 25.4 | 89 | 28.1 | 4 | 2.2 | 5 | 1.6 |  | 1.000 | 0.293 |  | 0.087 | 0.768 |  | 0.619 | 0.734 |
| S3 | A/G |  | 271 | 74.9 | 461 | 72.7 | 67 | 37.0 | 123 | 38.8 | 12 | 6.6 | 25 | 7.9 |  | 0.844 | 0.673 |  | 0.549 | 0.459 |  | 0.540 | 0.763 |
| S5 | T/C |  | 220 | 60.8 | 377 | 59.5 | 88 | 48.6 | 153 | 48.3 | 27 | 14.9 | 52 | 16.4 |  | 0.876 | 1.000 |  | 0.165 | 0.685 |  | 0.206 | 0.902 |
| S29 | T/C |  | 294 | 81.2 | 513 | 80.9 | 56 | 30.9 | 95 | 30.0 | 6 | 3.3 | 13 | 4.1 |  | 1.000 | 0.586 |  | 0.014 | 0.907 |  | 0.227 | 0.893 |

Allele and genotype association analysis comparing control group from Changsha (CON1) with control group from Beijing (CON2), China. Frequencies of the derived allele (D), heterozygous (D/N) and homozygous ancestral (N/N) allele genotypes were calculated for both control groups. Hardy-Weinberg equilibrium exact test (HWE) was performed for both control groups. *P*-value was computed by the likelihood ratio test.
