# Supplementary material for: GABRB2 Haplotype Association with Heroin Dependence in Chinese Population
Source: PLoS One. 2015 Nov 12;10(11):e0142049. doi: 10.1371/journal.pone.0142049 (PMC4643001; doi:10.1371/journal.pone.0142049)
Supplement: S4 Table — (DOCX) [file pone.0142049.s006.docx]

**S4 Table.** Association and linkage equilibrium analysis of individual SNPs

A. Allele association analysis for heroin dependence

| *SNP* |  | *Frequency* | | | | | | | |  | *HWE* | |  | *Allele* | | | |
| --- | --- | --- | --- | --- | --- | --- | --- | --- | --- | --- | --- | --- | --- | --- | --- | --- | --- |
|  |  | *D* | | | | *N* | | | |  |  |  |  |  |  |  |  |
|  |  | *HER* | | *CON* | | *HER* | | *CON* | |  | *HER* | *CON* |  | *OR* | *95% CI* | *χ^2^* | *P* |
|  |  | *n* | *%* | *n* | *%* | *n* | *%* | *n* | *%* |  |  |  |  |  |  |  |  |
| S31 |  | 26 | 3.4 | 6 | 2.1 | 742 | 69.6 | 280 | 97.9 |  | 1.000 | 1.000 |  | 0.61 | 0.25-1.50 | 1.264 | 0.261 |
| S32 |  | 33 | 13.5 | 32 | 16.0 | 211 | 86.5 | 168 | 84.0 |  | 1.000 | 0.711 |  | 1.22 | 0.72-2.06 | 0.537 | 0.464 |
| S1 |  | 961 | 85.2 | 843 | 84.6 | 167 | 14.8 | 153 | 15.4 |  | 0.615 | 0.394 |  | 0.96 | 0.75-1.22 | 0.128 | 0.720 |
| S3 |  | 830 | 73.6 | 732 | 73.5 | 298 | 26.4 | 264 | 26.5 |  | 0.588 | 0.646 |  | 1.00 | 0.82-1.21 | 0.002 | 0.964 |
| S5 |  | 722 | 64.0 | 597 | 59.9 | 406 | 36.0 | 399 | 40.1 |  | 0.648 | 0.926 |  | 0.84 | 0.70-1.00 | 3.716 | 0.054 |
| S29 |  | 890 | 78.9 | 807 | 81.0 | 238 | 21.1 | 189 | 19.0 |  | **0.016** | 0.770 |  | 1.14 | 0.92-1.41 | 1.488 | 0.222 |

1. Allele association analysis for heroin dependence comparing heroin dependent individuals (HER) with the combined control (CON) groups. Frequencies of the derived allele (D) and ancestral allele (N), and Hardy-Weinberg equilibrium exact test (HWE) were calculated for both sample groups. Odd ratios (OR) and 95% confidence interval (95% CI) of the ancestral allele are shown. *P*-value was computed by the likelihood ratio test. *P-*value < 0.05 is shown in bold font.

B. Genotype association analysis for heroin dependence

| *SNP* |  | *Frequency* | | | | | | | | | | | |  | *Genotype* | |
| --- | --- | --- | --- | --- | --- | --- | --- | --- | --- | --- | --- | --- | --- | --- | --- | --- |
|  |  | *D/D* | | | | *D/N* | | | | *N/N* | | | |  |  |  |
|  |  | *HER* | | *CON* | | *HER* | | *CON* | | *HER* | | *CON* | |  | *χ^2^* | *P* |
|  |  | *n* | *%* | *n* | *%* | *n* | *%* | *n* | *%* | *n* | *%* | *n* | *%* |  |  |  |
| S31 |  | 0 | 0.0 | 0 | 0.0 | 26 | 6.8 | 6 | 4.2 | 358 | 93.2 | 137 | 95.8 |  | 1.302 | 0.254 |
| S32 |  | 2 | 1.6 | 3 | 3.0 | 29 | 23.8 | 26 | 26.0 | 91 | 74.6 | 71 | 71.0 |  | 0.657 | 0.720 |
| S1 |  | 411 | 72.9 | 354 | 71.1 | 139 | 24.6 | 135 | 27.1 | 14 | 2.5 | 9 | 1.8 |  | 1.301 | 0.522 |
| S3 |  | 308 | 54.6 | 271 | 54.4 | 214 | 37.9 | 190 | 38.2 | 42 | 7.5 | 37 | 7.4 |  | 0.005 | 0.998 |
| S5 |  | 228 | 40.4 | 178 | 35.7 | 266 | 47.2 | 241 | 48.4 | 70 | 12.4 | 79 | 15.9 |  | 3.846 | 0.146 |
| S29 |  | 361 | 64.0 | 328 | 65.9 | 168 | 29.8 | 151 | 30.3 | 35 | 6.2 | 19 | 3.8 |  | 3.196 | 0.202 |

1. Genotype association analysis with heroin dependence comparing heroin dependent individuals with the combined control groups. Frequencies of homozygous derived (D/D), heterozygous (D/N), and homozygous ancestral (N/N) allele genotypes were calculated for both sample groups. *P*-value was computed by the likelihood ratio test.

C. Values of the linkage disequilibrium coefficients *D’* and *r^2^* for all pairwise SNP pairs in the combined control group (CON).

| *SNP pairs* | | *D’* | *r^2^* |
| --- | --- | --- | --- |
| S31 | S32 | 1.000 | 0.006 |
| S31 | S1 | 1.000 | 0.004 |
| S31 | S3 | 1.000 | 0.060 |
| S31 | S5 | 1.000 | 0.034 |
| S31 | S29 | 1.000 | 0.090 |
| S32 | S1 | 0.866 | 0.024 |
| S32 | S3 | 1.000 | 0.065 |
| S32 | S5 | 1.000 | 0.122 |
| S32 | S29 | 1.000 | 0.042 |
| S1 | S3 | 0.970 | 0.473 |
| S1 | S5 | 0.985 | 0.264 |
| S1 | S29 | 0.932 | 0.674 |
| S3 | S5 | 0.985 | 0.524 |
| S3 | S29 | 0.929 | 0.561 |
| S5 | S29 | 0.955 | 0.320 |
