# Supplementary material for: GABRB2 Haplotype Association with Heroin Dependence in Chinese Population
Source: PLoS One. 2015 Nov 12;10(11):e0142049. doi: 10.1371/journal.pone.0142049 (PMC4643001; doi:10.1371/journal.pone.0142049)
Supplement: S5 Table — (DOCX) [file pone.0142049.s007.docx]

**S5 Table.** Quantitative trait analysis on duration of heroin dependence

| *SNP* |  | *Female + Male* | |  | *Female* | |  | *Male* | |
| --- | --- | --- | --- | --- | --- | --- | --- | --- | --- |
|  |  | *Effect size* | *P* |  | *Effect size* | *P* |  | *Effect size* | *P* |
| S1 |  | 0.048 | 0.259 |  | 0.096 | 0.202 |  | 0.019 | 0.709 |
| S3 |  | 0.012 | 0.768 |  | 0.054 | 0.479 |  | 0.011 | 0.821 |
| S5 |  | 0.021 | 0.611 |  | 0.015 | 0.844 |  | 0.024 | 0.640 |
| S29 |  | 0.028 | 0.513 |  | 0.117 | 0.120 |  | 0.027 | 0.595 |

Quantitative trait analysis of genotype distribution on duration of heroin dependence in female and male samples. Effect size is represented by the Pearson’s *r* value and *P*-value was calculated by the linear-by-linear association test.
