# Supplementary material for: GABRB2 Haplotype Association with Heroin Dependence in Chinese Population
Source: PLoS One. 2015 Nov 12;10(11):e0142049. doi: 10.1371/journal.pone.0142049 (PMC4643001; doi:10.1371/journal.pone.0142049)
Supplement: S6 Table — (DOCX) [file pone.0142049.s008.docx]

**S6 Table.** Four-SNPs haplotype association analysis for heroin dependence

(A) Female + Male

| *Haplotype* | *Hap No.* |  | *HER*  *Frequency* | | *CON Frequency* | |  | *OR* | *95% CI* | *χ2* | *P* | *P_Global_* |
| --- | --- | --- | --- | --- | --- | --- | --- | --- | --- | --- | --- | --- |
|  |  |  | *n* | *%* | *n* | *%* |  |  |  |  |  |  |
| D-D-D-D | H1 |  | 665 | 58.9 | 588 | 59.0 |  | 1.00 | 1.00-1.00 | 0.023 | 0.878 | ─ |
| N-N-N-N | H2 |  | 120 | 10.7 | 144 | 14.4 |  | 0.74 | 0.57-0.97 | 5.569 | **0.018** | **0.0183** |
| D-D-D-N | H3 |  | 48 | 4.3 | 5 | 0.5 |  | 8.51 | 3.04-23.83 | 28.080 | **1.164x10^-7^** | **0.0001** |
| N-N-N-D | H4 |  | 39 | 3.4 | 6 | 0.6 |  | 5.58 | 2.28-13.64 | 16.900 | **3.945x10^-5^** | **0.0001** |
| N-D-D-N | H5 |  | 5 | 0.5 | 0 | 0.0 |  | 1.94x10^9^ | 1.19x10^9^-3.18x10^9^ | 4.035 | **0.044** | **0.0460** |
| D-D-N-D | H6 |  | 102 | 9.0 | 132 | 13.3 |  | 0.68 | 0.51-0.91 | 9.929 | **0.002** | **0.0026** |
| D-D-N-N | ─ |  | 7 | 0.6 | 4 | 0.4 |  | 1.71 | 0.39-7.58 | 1.510 | 0.219 | ─ |
| D-N-N-N | ─ |  | 55 | 4.9 | 36 | 3.6 |  | 1.36 | 0.86-2.14 | 2.532 | 0.112 | ─ |
| D-N-N-D | ─ |  | 81 | 7.1 | 76 | 7.7 |  | 0.93 | 0.66-1.32 | 0.317 | 0.574 | ─ |
| D-N-D-N | ─ |  | 2 | 0.2 | 0 | 0.0 |  | 11.52 | 4.42x10^-8^-3.00x10^9^ | 0.651 | 0.420 | ─ |
| D-N-D-D | ─ |  | 1 | 0.1 | 2 | 0.2 |  | 0.50 | 0.03-8.33 | 0.081 | 0.776 | ─ |
| N-D-N-N | ─ |  | 1 | 0.1 | 1 | 0.1 |  | 0.06 | 0.00-1.41x10^11^ | 0.191 | 0.662 | ─ |
| N-D-N-D | ─ |  | 2 | 0.2 | 0 | 0.0 |  | 3.11 | 0.10-94.74 | 0.336 | 0.562 | ─ |
| N-D-D-D | ─ |  | 0 | 0.0 | 2 | 0.2 |  | 2.17x10^-8^ | 2.17x10^-8^-2.17x10^-8^ | 0.993 | 0.319 | ─ |

(B) Male

| *Haplotype* | *Hap No.* |  | *HER*  *Frequency* | | *CON Frequency* | |  | *OR* | *95% CI* | *χ2* | *P* | *P_Global_* |
| --- | --- | --- | --- | --- | --- | --- | --- | --- | --- | --- | --- | --- |
|  |  |  | *n* | *%* | *n* | *%* |  |  |  |  |  |  |
| D-D-D-D | H1 |  | 468 | 60.4 | 328 | 56.0 |  | 1.00 | 1.00-1.00 | 3.271 | 0.070 | ─ |
| N-N-N-N | H2 |  | 78 | 10.0 | 97 | 16.5 |  | 0.56 | 0.40-0.79 | 10.860 | **9.815x10^-4^** | **0.0010** |
| D-D-D-N | H3 |  | 32 | 4.1 | 0 | 0.0 |  | 2.08x10^9^ | 1.73x10^9^-2.54x10^9^ | 20.540 | **5.842x10^-6^** | **0.0001** |
| N-N-N-D | H4 |  | 30 | 3.8 | 1 | 0.2 |  | 19.91 | 2.69-147.30 | 17.000 | **3.738x10^-5^** | **0.0001** |
| N-D-D-N | H5 |  | 2 | 0.3 | 0 | 0.0 |  | 3.40x10^10^ | 1.70x10^10^-6.82x10^10^ | 1.695 | 0.193 | ─ |
| D-D-N-D | H6 |  | 72 | 9.2 | 82 | 14.0 |  | 0.61 | 0.43-0.87 | 8.215 | **0.004** | **0.0049** |
| D-D-N-N | ─ |  | 4 | 0.5 | 3 | 0.6 |  | 0.76 | 0.14-4.07 | 0.110 | 0.740 | ─ |
| D-N-N-N | ─ |  | 34 | 4.4 | 16 | 2.8 |  | 1.49 | 0.79-2.80 | 2.638 | 0.104 | ─ |
| D-N-N-D | ─ |  | 52 | 6.7 | 52 | 9.0 |  | 0.69 | 0.46-1.05 | 2.538 | 0.111 | ─ |
| D-N-D-N | ─ |  | 1 | 0.2 | 1 | 0.2 |  | 1.15 | 0.01-90.36 | 0.002 | 0.968 | ─ |
| D-N-D-D | ─ |  | 0 | 0.0 | 1 | 0.2 |  | 3.89x10^-8^ | 3.89x10^-8^-3.89x10^-8^ | 2.194 | 0.139 | ─ |
| N-D-N-N | ─ |  | 1 | 0.1 | 1 | 0.2 |  | 0.32 | 2.81x10^-3^-37.73 | 0.182 | 0.670 | ─ |
| N-D-N-D | ─ |  | 2 | 0.3 | 1 | 0.1 |  | 2.51 | 0.08-77.39 | 0.232 | 0.630 | ─ |
| N-D-D-D | ─ |  | 0 | 0.0 | 1 | 0.2 |  | 0.00 | 0.00-0.00 | 1.059 | 0.303 | ─ |

Four-SNPs haplotype association analysis comparing heroin dependent (HER) with the combined control (CON) groups. Frequency of four-SNPs haplotypes is separately analyzed in female + male (Part A) (case n = 564; control n = 498) and male (Part B) (case n = 338; control n = 293) samples. N refers to the ancestral allele and D refers to the derived allele. Odd ratios (OR) and 95% confidence interval (95% CI) are based on the all-derived-allele haplotype (H1 haplotype). Odds ratio of 1.00 serves as the baseline for determining haplotype risks. Haplotypes with odds ratios higher than 1.00 are considered as risk haplotypes and those with odds ratios lower than 1.00 are considered as protective haplotypes. *P*-value was computed by the likelihood ratio test and significant P values (*P* < 0.05; *P_Global_* < 0.05) are in bold font. Detailed analysis of H1-H6 haplotypes for susceptibility to heroin dependence is shown in Fig 2.
