# Supplementary material for: GABRB2 Haplotype Association with Heroin Dependence in Chinese Population
Source: PLoS One. 2015 Nov 12;10(11):e0142049. doi: 10.1371/journal.pone.0142049 (PMC4643001; doi:10.1371/journal.pone.0142049)
Supplement: S7 Table — (DOCX) [file pone.0142049.s009.docx]

**S7 Table.** Six-SNPs haplotype association analysis for heroin dependence

(A) Female + Male

| *Haplotype* | *Hap No.* |  | *HER*  *Frequency* | | *CON Frequency* | |  | *OR* | *95% CI* | *χ2* | *P* | *P_Global_* |
| --- | --- | --- | --- | --- | --- | --- | --- | --- | --- | --- | --- | --- |
|  |  |  | *n* | *%* | *n* | *%* |  |  |  |  |  |  |
| N-N-D-D-D-D | H1 |  | 66 | 41.1 | 77 | 45.4 |  | 1.00 | 1.00-1.00 | 0.189 | 0.664 | ─ |
| D-D-N-N-N-N | H2 |  | 0 | 0.0 | 1 | 0.6 |  | 0.00 | 0.00-0.00 | 0.920 | 0.337 | ─ |
| N-N-D-D-D-N | H3 |  | 16 | 9.9 | 1 | 0.6 |  | 18.39 | 2.24-151.00 | 11.600 | **6.595x10^-4^** | **0.0015** |
| N-N-N-N-N-D | H4 |  | 13 | 8.6 | 2 | 1.0 |  | 9.76 | 1.15-82.58 | 8.500 | **0.004** | **0.0043** |
| N-N-N-D-D-N | H5 |  | 3 | 1.7 | 0 | 0.0 |  | 1.58x10^12^ | 7.72x10^11^-3.24x10^12^ | 4.254 | **0.039** | 0.0718 |
| N-N-D-D-N-D | H6 |  | 12 | 7.7 | 21 | 12.4 |  | 0.68 | 0.29-1.59 | 1.582 | 0.208 | ─ |
| D-N-D-N-N-D | H7 |  | 5 | 3.1 | 0 | 0.0 |  | 1.45x10^11^ | 8.59x10^10^-2.43x10^11^ | 4.116 | **0.042** | **0.0392** |

(B) Male

| *Haplotype* | *Hap No.* |  | *HER*  *Frequency* | | *CON Frequency* | |  | *OR* | *95% CI* | *χ2* | *P* | *P_Global_* |
| --- | --- | --- | --- | --- | --- | --- | --- | --- | --- | --- | --- | --- |
|  |  |  | *n* | *%* | *n* | *%* |  |  |  |  |  |  |
| N-N-D-D-D-D | H1 |  | 58 | 39.5 | 58 | 47.4 |  | 1.00 | 1.00-1.00 | 0.884 | 0.347 | ─ |
| D-D-N-N-N-N | H2 |  | 0 | 0.0 | 1 | 0.8 |  | 0.00 | 0.00-0.00 | 1.218 | 0.270 | ─ |
| N-N-D-D-D-N | H3 |  | 16 | 11.0 | 0 | 0.0 |  | 1.04x10^19^ | 7.50x10^18^-1.44x10^19^ | 11.560 | **6.743x10^-4^** | **0.0004** |
| N-N-N-N-N-D | H4 |  | 13 | 8.6 | 0 | 0.0 |  | 1.72x10^19^ | 1.20x10^19^-2.46x10^19^ | 9.056 | **0.003** | **0.0012** |
| N-N-N-D-D-N | H5 |  | 3 | 1.8 | 0 | 0.0 |  | 3.98x10^18^ | 1.94x10^18^-8.16x10^18^ | 2.657 | 0.103 | ─ |
| N-N-D-D-N-D | H6 |  | 12 | 8.2 | 13 | 10.8 |  | 0.91 | 0.36-2.29 | 0.390 | 0.532 | ─ |
| D-N-D-N-N-D | H7 |  | 5 | 3.4 | 0 | 0.0 |  | 1.14x10^18^ | 6.78x10^17^-1.92x10^18^ | 3.139 | 0.076 | ─ |

Six-SNPs haplotype association analysis comparing heroin dependent group (HER) with the combined control group (CON). Frequency of six-SNPs haplotypes was separately analyzed in female + male (Part A) (case n = 564; control n = 498) and male (Part B) (case n = 388; control n = 293) samples. N refers to the ancestral allele and D refers to the derived allele. Odd ratios (OR) and 95% confidence interval (95% CI) are based on the major haplotype with the ancestral alleles for S31 and S32 and derived alleles for S1, S3, S5, and S29 (H1 haplotype). Odds ratio of 1.00 serves as the baseline for determining haplotype risks. Haplotypes with odds ratios higher than 1.00 are considered as risk haplotypes and those with odds ratio lower than 1.00 are considered as protective haplotypes. *P*-value was computed by the likelihood ratio test and significant *P*-values (*P* < 0.05; *P_Global_* < 0.05) are in bold font. Only haplotypes analyzed in S1 Figure (H1-H7 haplotypes) are shown in this table.
