# Supplementary material for: GABRB2 Haplotype Association with Heroin Dependence in Chinese Population
Source: PLoS One. 2015 Nov 12;10(11):e0142049. doi: 10.1371/journal.pone.0142049 (PMC4643001; doi:10.1371/journal.pone.0142049)
Supplement: S8 Table — (DOCX) [file pone.0142049.s010.docx]

**S8 Table.** Multiple-SNPs haplotype association analysis for heroin dependence in substance dependence subgroups

(A) Overall heroin dependent group (HER)

| *Haplotype marker combinations* | | | | *Haplotype* |  | *Case Frequency* | | *Control Frequency* | |  | *OR* | *95% CI* | *χ2* | *P* | *P_Global_* |
| --- | --- | --- | --- | --- | --- | --- | --- | --- | --- | --- | --- | --- | --- | --- | --- |
| *S1* | *S3* | *S5* | *S29* |  |  | *n* | *%* | *n* | *%* |  |  |  |  |  |  |
| X | X | X | X | N-D-D-N |  | 5 | 0.5 | 0 | 0.0 |  | 1.94x10^9^ | 1.19x10^9^-3.18x10^9^ | 4.035 | **0.044** | **0.0460** |
|  |  | X | X | D-N |  | 58 | 5.2 | 5 | 0.5 |  | 10.22 | 3.64-28.64 | 31.070 | **2.495x10^-8^** | **0.0001** |
|  | X | X | X | D-D-N |  | 54 | 4.8 | 5 | 0.5 |  | 9.62 | 0.34-26.81 | 30.690 | **3.028x10^-8^** | **0.0001** |
| X |  | X | X | D-D-N |  | 52 | 4.6 | 5 | 0.5 |  | 9.48 | 3.38-26.59 | 30.910 | **2.698x10^-8^** | **0.0001** |
| X | X | X | X | D-D-D-N |  | 48 | 4.3 | 5 | 0.5 |  | 8.51 | 3.04-23.83 | 28.080 | **1.164x10^-7^** | **0.0001** |
| X | X |  | X | D-D-N |  | 55 | 4.9 | 8 | 0.8 |  | 6.09 | 2.86-12.99 | 27.220 | **1.816x10^-7^** | **0.0001** |
| X | X |  | X | N-N-D |  | 39 | 3.4 | 6 | 0.6 |  | 5.96 | 2.43-14.62 | 16.940 | **3.851x10^-5^** | **0.0001** |
|  | X |  | X | D-N |  | 61 | 5.4 | 10 | 1.0 |  | 5.88 | 2.88-12.00 | 27.960 | **1.237x10^-7^** | **0.0001** |
| X | X | X | X | N-N-N-D |  | 39 | 3.4 | 6 | 0.6 |  | 5.58 | 2.28-13.64 | 16.900 | **3.945x10^-5^** | **0.0001** |
| X |  | X | X | N-N-D |  | 42 | 3.7 | 7 | 0.7 |  | 5.37 | 2.26-12.77 | 16.990 | **3.754x10^-5^** | **0.0001** |
| X |  |  | X | N-D |  | 42 | 3.7 | 8 | 0.8 |  | 4.73 | 2.19-10.21 | 15.690 | **7.466x10^-5^** | **0.0001** |
| X |  |  | X | D-N |  | 113 | 10.0 | 44 | 4.4 |  | 2.40 | 1.67-3.46 | 21.330 | **3.868x10^-6^** | **0.0002** |
| X |  |  | X | D-D |  | 848 | 75.2 | 799 | 80.2 |  | 1.00 | 1.00-1.00 | 6.556 | **0.010** | **0.0135** |
| X |  |  | X | N-N |  | 125 | 11.1 | 145 | 14.5 |  | 0.81 | 0.63-1.06 | 4.525 | **0.033** | **0.0400** |
| X | X |  | X | N-N-N |  | 120 | 10.7 | 144 | 14.4 |  | 0.79 | 0.60-1.03 | 5.578 | **0.018** | **0.0183** |
| X |  | X | X | D-N-D |  | 183 | 16.2 | 208 | 20.9 |  | 0.78 | 0.62-0.99 | 8.469 | **0.004** | **0.0045** |
| X |  | X | X | N-N-N |  | 120 | 10.7 | 144 | 14.5 |  | 0.74 | 0.56-0.97 | 5.625 | **0.018** | **0.0191** |
| X | X | X | X | N-N-N-N |  | 120 | 10.7 | 145 | 14.4 |  | 0.74 | 0.57-0.97 | 5.569 | **0.018** | **0.0183** |
|  | X | X | X | D-N-D |  | 104 | 9.2 | 132 | 13.3 |  | 0.70 | 0.52-0.92 | 9.362 | **0.002** | **0.0037** |
| X | X | X | X | D-D-N-D |  | 102 | 9.0 | 132 | 13.3 |  | 0.68 | 0.51-0.91 | 9.929 | **0.002** | **0.0026** |
|  | X | X |  | D-N |  | 108 | 9.9 | 135 | 13.8 |  | 0.67 | 0.51-0.88 | 7.856 | **0.005** | **0.0068** |
| X | X | X |  | D-D-N |  | 111 | 9.6 | 137 | 13.6 |  | 0.67 | 0.51-0.88 | 8.176 | **0.004** | **0.0067** |

1. Pure heroin dependence subgroup

| *Haplotype marker combinations* | | | | *Haplotype* |  | *Case Frequency* | | *Control Frequency* | |  | *OR* | *95% CI* | *χ2* | *P* | *P_Global_* |
| --- | --- | --- | --- | --- | --- | --- | --- | --- | --- | --- | --- | --- | --- | --- | --- |
| *S1* | *S3* | *S5* | *S29* |  |  | *n* | *%* | *n* | *%* |  |  |  |  |  |  |
|  |  | X | X | D-N |  | 21 | 4.2 | 5 | 0.5 |  | 8.16 | 2.71-24.58 | 21.140 | **4.267x10^-6^** | **0.0001** |
| X |  | X | X | D-D-N |  | 19 | 3.9 | 5 | 0.5 |  | 7.79 | 2.58-23.54 | 21.600 | **3.351x10^-6^** | **0.0001** |
|  | X | X | X | D-D-N |  | 18 | 3.7 | 5 | 0.5 |  | 7.30 | 2.42-21.95 | 19.070 | **1.258x10^-5^** | **0.0001** |
| X | X | X | X | D-D-D-N |  | 17 | 3.5 | 5 | 0.5 |  | 6.69 | 2.21-20.30 | 18.140 | **2.055x10^-5^** | **0.0001** |
| X | X |  | X | D-D-N |  | 21 | 4.2 | 8 | 0.8 |  | 5.14 | 2.22-11.91 | 17.840 | **2.406x10^-5^** | **0.0004** |
|  | X |  | X | D-N |  | 22 | 4.5 | 10 | 1.0 |  | 4.74 | 2.13-10.55 | 16.780 | **4.192x10^-5^** | **0.0005** |
| X | X |  | X | N-N-D |  | 13 | 2.6 | 6 | 0.6 |  | 4.40 | 1.60-12.13 | 8.812 | **0.003** | **0.0081** |
| X | X | X | X | N-N-N-D |  | 13 | 2.6 | 6 | 0.6 |  | 4.12 | 1.50-11.32 | 8.745 | **0.003** | **0.0088** |
| X |  | X | X | N-N-D |  | 14 | 2.8 | 7 | 0.7 |  | 4.05 | 1.53-10.76 | 8.939 | **0.003** | **0.0063** |
| X |  |  | X | N-D |  | 14 | 2.9 | 8 | 0.8 |  | 3.55 | 1.46-8.66 | 7.681 | **0.006** | **0.0074** |
| X |  |  | X | D-N |  | 43 | 8.8 | 44 | 4.4 |  | 2.05 | 1.32-3.78 | 10.100 | **0.001** | **0.0043** |
| X |  | X | X | D-N-D |  | 82 | 16.7 | 208 | 20.9 |  | 0.79 | 0.58-1.06 | 4.087 | **0.043** | **0.0429** |
|  | X | X | X | D-N-D |  | 46 | 9.4 | 132 | 13.3 |  | 0.70 | 0.48-1.00 | 4.788 | **0.029** | **0.0294** |
| X | X | X | X | D-D-N-D |  | 45 | 9.2 | 132 | 13.3 |  | 0.68 | 0.47-0.99 | 5.074 | **0.024** | **0.0246** |

1. Multiple substance dependent subgroup

| *Haplotype marker combinations* | | | | *Haplotype* |  | *Case Frequency* | | *Control Frequency* | |  | *OR* | *95% CI* | *χ2* | *P* | *P_Global_* |
| --- | --- | --- | --- | --- | --- | --- | --- | --- | --- | --- | --- | --- | --- | --- | --- |
| *S1* | *S3* | *S5* | *S29* |  |  | *n* | *%* | *n* | *%* |  |  |  |  |  |  |
| X | X | X | X | N-D-D-N |  | 4 | 0.6 | 0 | 0.0 |  | 3.86x10^10^ | 2.18x10^10^-6.82x10^10^ | 5.910 | **0.015** | **0.0087** |
| X |  | X | X | N-D-N |  | 4 | 0.6 | 0 | 0.0 |  | 2.56x10^8^ | 1.45x10^8^-4.52x10^8^ | 4.586 | **0.032** | **0.0373** |
|  |  | X | X | D-N |  | 38 | 5.9 | 5 | 0.5 |  | 11.93 | 4.15-34.33 | 34.760 | **3.724x10^-9^** | **0.0001** |
|  | X | X | X | D-D-N |  | 36 | 5.7 | 5 | 0.5 |  | 11.73 | 4.11-33.50 | 36.490 | **1.537x10^-9^** | **0.0001** |
| X |  | X | X | D-D-N |  | 33 | 5.2 | 5 | 0.5 |  | 10.88 | 3.77-31.42 | 34.700 | **3.839x10^-9^** | **0.0001** |
| X | X | X | X | D-D-D-N |  | 32 | 5.0 | 5 | 0.5 |  | 10.13 | 3.52-29.17 | 32.430 | **1.233x10^-8^** | **0.0001** |
| X | X |  | X | N-N-D |  | 26 | 4.1 | 6 | 0.6 |  | 7.24 | 2.86-18.33 | 20.230 | **6.857x10^-6^** | **0.0001** |
| X | X |  | X | D-D-N |  | 34 | 5.4 | 8 | 0.8 |  | 6.87 | 3.11-15.16 | 29.000 | **7.245x10^-8^** | **0.0001** |
|  | X |  | X | D-N |  | 39 | 6.2 | 10 | 10. |  | 6.81 | 3.23-14.36 | 30.790 | **2.875x10^-8^** | **0.0001** |
| X | X | X | X | N-N-N-D |  | 26 | 4.1 | 6 | 0.6 |  | 6.78 | 2.69-17.12 | 20.180 | **7.040x10^-6^** | **0.0001** |
| X |  | X | X | N-N-D |  | 28 | 4.4 | 7 | 0.7 |  | 6.47 | 2.63-15.90 | 20.000 | **7.747x10^-6^** | **0.0001** |
| X |  |  | X | N-D |  | 28 | 4.4 | 8 | 0.8 |  | 5.72 | 2.55-12.81 | 18.460 | **1.733x10^-5^** | **0.0002** |
| X |  |  | X | D-N |  | 70 | 11.0 | 44 | 4.4 |  | 2.69 | 1.81-4.01 | 22.550 | **2.049x10^-6^** | **0.0001** |
| X |  |  | X | D-D |  | 469 | 73.7 | 799 | 80.2 |  | 1.00 | 1.00-1.00 | 8.015 | **0.005** | **0.0062** |
|  | X |  | X | D-D |  | 426 | 66.9 | 722 | 72.5 |  | 1.00 | 1.00-1.00 | 4.604 | **0.032** | **0.0338** |
| X | X |  | X | D-D-D |  | 425 | 66.8 | 720 | 72.3 |  | 1.00 | 1.00-1.00 | 4.746 | **0.029** | **0.0311** |
| X |  | X | X | D-N-D |  | 101 | 15.9 | 208 | 20.9 |  | 0.78 | 0.59-1.03 | 7.175 | **0.007** | **0.0082** |
| X | X |  | X | N-N-N |  | 65 | 10.2 | 144 | 14.4 |  | 0.77 | 0.56-1.06 | 4.747 | **0.029** | **0.0259** |
| X | X | X | X | N-N-N-N |  | 65 | 10.3 | 144 | 14.4 |  | 0.73 | 0.52-1.00 | 4.737 | **0.030** | **0.0259** |
| X |  | X | X | N-N-N |  | 65 | 10.3 | 145 | 14.5 |  | 0.72 | 0.52-1.00 | 4.609 | **0.032** | **0.0317** |
|  | X | X | X | D-N-D |  | 58 | 9.1 | 132 | 13.3 |  | 0.70 | 0.50-0.99 | 7.189 | **0.007** | **0.0094** |
| X | X | X | X | D-D-N-D |  | 57 | 8.9 | 132 | 13.3 |  | 0.69 | 0.49-0.97 | 7.602 | **0.006** | **0.0072** |
|  | X | X |  | D-N |  | 61 | 9.6 | 137 | 13.8 |  | 0.66 | 0.47-0.91 | 6.309 | **0.012** | **0.0149** |
| X | X | X |  | D-D-N |  | 59 | 9.3 | 135 | 13.6 |  | 0.65 | 0.46-0.90 | 6.730 | **0.009** | **0.0115** |

Haplotype frequencies of overall heroin dependent group (HER) (Part A) (n = 564), pure heroin dependent (Part B) (n = 246) and multiple substance (Part C) (n = 318) subgroups were compared with haplotype frequencies of the combined control (CON) group (n = 498). Each SNP included in a haplotype association test is denoted by ‘X’. N refers to the ancestral allele and D refers to the derived allele. Odd ratios (OR) and 95% confidence interval (95% CI) are based on the homozygous derived allele haplotype. Odds ratio of 1.00 serves as the baseline for determining haplotype risks. Haplotypes with odds ratios higher than 1.00 are considered as risk haplotypes and those with odds ratio lower than 1.00 are considered as protective haplotypes. *P*-value was computed by the likelihood ratio test. Only haplotypes maintaining significant association with heroin dependence after the global permutation test (*P* < 0.05; *P_Global_* < 0.05) are shown in the tables and significant *P-*values are in bold font.
