# Supplementary material for: GABRB2 Haplotype Association with Heroin Dependence in Chinese Population
Source: PLoS One. 2015 Nov 12;10(11):e0142049. doi: 10.1371/journal.pone.0142049 (PMC4643001; doi:10.1371/journal.pone.0142049)
Supplement: S1 Text — (DOCX) [file pone.0142049.s011.docx]

**S1 Text.** R-script for samples resampling.

data_fn <- 'c:/Users/user/Desktop/Resampling/genotype.txt'

do_resampling <- function(data_fn, resample_n=1000, resample_size=218)

{

# Check if the data file exists

if(!file.exists(data_fn))

{

warning(paste(data_fn, " is not found.", sep=""));

stop();

}

cat(paste("Importing datafile: ", data_fn, "\n",sep=""));

set.seed(as.integer(unclass(as.POSIXlt(Sys.time()))$sec * 10000));

# Import the data

data <- read.table(data_fn, sep="\t", header=T, stringsAsFactors=F);

case_males <- which(data$Sex == 1 & data$Disease.status == 2);

case_females <- which(data$Sex == 2 & data$Disease.status == 2);

control_males <- which(data$Sex == 1 & data$Disease.status == 1);

control_females <- which(data$Sex == 2 & data$Disease.status == 1);

if(length(case_males) == 0)

{

warning("No case found for Sex=1 and Disease.status=2");

stop();

}

if(length(case_females) == 0)

{

warning("No case found for Sex=2 and Disease.status=2");

stop();

}

if(length(control_males) == 0)

{

warning("No case found for Sex=1 and Disease.status=1");

stop();

}

if(length(control_females) == 0)

{

warning("No case found for Sex=2 and Disease.status=1");

stop();

}

cat("Summary:\n");

cat("CASE (Disease Status == 2)\n");

cat("Male (1) Female (2)\n");

cat(paste(length(case_males), " ", length(case_females), "\n", sep=""));

cat("CONTROL (Disease Status == 1)\n");

cat("Male (1) Female (2)\n");

cat(paste(length(control_males), " ", length(control_females), "\n", sep=""));

# Resample data

for(i in 1 : resample_n)

{

cat(paste("Resampling round: ", i, "\n", sep=""));

# Set random seed

set.seed(as.integer(unclass(as.POSIXlt(Sys.time()))$sec * 10000));

resample_fn <- paste(data_fn, ".resample_", i, sep="", ".txt");

#

resample_case_males <- sample(case_males, resample_size, replace=F);

resample_case_females <- sample(case_females, resample_size, replace=F);

resample_control_males <- sample(control_males, resample_size, replace=F);

resample_control_females <- sample(control_females, resample_size, replace=F);

# Create a new resample data

resample_data <- as.data.frame(setNames(replicate(ncol(data), character(0), simplify=F),colnames(data)));

#

resample_data <- rbind(resample_data , data[resample_case_males,]);

resample_data <- rbind(resample_data , data[resample_case_females,]);

resample_data <- rbind(resample_data , data[resample_control_males,]);

resample_data <- rbind(resample_data , data[resample_control_females,]);

cat(paste("Exporting resampling result to ", resample_fn, "\n", sep=""))

write.table(resample_data, file=resample_fn, quote=F, sep="\t", col.names=F, row.names=F);

}

cat(paste("Completed!\n", sep=""))

}
